# Dynamics of Chromosome Replication and Its Relationship to Predatory Attack Lifestyles in Bdellovibrio bacteriovorus

**DOI:** 10.1128/AEM.00730-19

**Published:** 2019-07-01

**Authors:** Łukasz Makowski, Damian Trojanowski, Rob Till, Carey Lambert, Rebecca Lowry, R. Elizabeth Sockett, Jolanta Zakrzewska-Czerwińska

**Affiliations:** a Department of Microbiology, Hirszfeld Institute of Immunology and Experimental Therapy, Polish Academy of Sciences, Wroclaw, Poland; b Department of Molecular Microbiology, Faculty of Biotechnology, University of Wroclaw, Wroclaw, Poland; c School of Life Sciences, Queen's Medical Centre, University of Nottingham, Nottingham, United Kingdom; University of Tartu

**Keywords:** DNA replication, DnaN, *Bdellovibrio*, chromosome replication dynamics, predatory bacterium, replisome

## Abstract

Bdellovibrio bacteriovorus is a small Gram-negative, obligate predatory bacterium that is largely found in wet, aerobic environments (e.g., soil). This bacterium attacks and invades other Gram-negative bacteria, including animal and plant pathogens. The intriguing life cycle of B. bacteriovorus consists of two phases: a free-living nonreplicative attack phase, in which the predatory bacterium searches for its prey, and a reproductive phase, in which B. bacteriovorus degrades a host’s macromolecules and reuses them for its own growth and chromosome replication. Although the cell biology of this predatory bacterium has gained considerable interest in recent years, we know almost nothing about the dynamics of its chromosome replication. Here, we performed a real-time investigation into the subcellular localization of the replisome(s) in single cells of B. bacteriovorus. Our results show that in B. bacteriovorus, chromosome replication takes place only during the reproductive phase and exhibits a novel spatiotemporal arrangement of replisomes. The replication process starts at the invasive pole of the predatory bacterium inside the prey cell and proceeds until several copies of the chromosome have been completely synthesized. Chromosome replication is not coincident with the predator cell division, and it terminates shortly before synchronous predator filament septation occurs. In addition, we demonstrate that if this B. bacteriovorus life cycle fails in some cells of Escherichia coli, they can instead use second prey cells to complete their life cycle.

**IMPORTANCE** New strategies are needed to combat multidrug-resistant bacterial infections. Application of the predatory bacterium Bdellovibrio bacteriovorus, which kills other bacteria, including pathogens, is considered promising for combating bacterial infections. The B. bacteriovorus life cycle consists of two phases, a free-living, invasive attack phase and an intracellular reproductive phase, in which this predatory bacterium degrades the host’s macromolecules and reuses them for its own growth. To understand the use of B. bacteriovorus as a “living antibiotic,” it is first necessary to dissect its life cycle, including chromosome replication. Here, we present a real-time investigation into subcellular localization of chromosome replication in a single cell of B. bacteriovorus. This process initiates at the invasion pole of B. bacteriovorus and proceeds until several copies of the chromosome have been completely synthesized. Interestingly, we demonstrate that some cells of B. bacteriovorus require two prey cells sequentially to complete their life cycle.

## Abstract (precis)

New strategies are needed to combat multidrug-resistant bacterial infections. Application of the predatory bacterium Bdellovibrio bacteriovorus, which kills other bacteria, including pathogens, is considered promising for combating bacterial infections. The B. bacteriovorus life cycle consists of two phases, a free-living, invasive attack phase and an intracellular reproductive phase, in which this predatory bacterium degrades the host’s macromolecules and reuses them for its own growth. To understand the use of B. bacteriovorus as a “living antibiotic,” it is first necessary to dissect its life cycle, including chromosome replication. Here, we present a real-time investigation into subcellular localization of chromosome replication in a single cell of B. bacteriovorus. This process initiates at the invasion pole of B. bacteriovorus and proceeds until several copies of the chromosome have been completely synthesized. Interestingly, we demonstrate that some cells of B. bacteriovorus require two prey cells sequentially to complete their life cycle.

## INTRODUCTION

Bdellovibrio bacteriovorus is a small (0.2 to 0.5 μm wide and 0.5 to 2.5 μm long) Gram-negative bacterium that is unusual in its ability to invade and kill other Gram-negative bacteria. Moreover, it was demonstrated that B. bacteriovorus also benefits from interacting with Gram-positive biofilms (Staphylococcus aureus) (1). Bacteria belonging to the genus Bdellovibrio are largely found in wet, aerobic environments (e.g., soil) (2). B. bacteriovorus has received considerable research interest due to its intriguing life cycle and its great potential to be applied as an antimicrobial agent in industry, agriculture, and medicine. This bacterium proliferates within the periplasm of the prey cell and can invade a wide range of bacteria, including plant and animal pathogens (3–8).

B. bacteriovorus has a biphasic life cycle (see Fig. 5) that consists of (i) a nongrowing attack phase, in which a predatory bacterium finds a prey cell, attaches to its outer membrane and enters the periplasm; and (ii) a reproductive phase, in which B. bacteriovorus degrades the host’s macromolecules and reuses them for its own growth and chromosome replication. During the attack phase, B. bacteriovorus actively seeks the prey cell and is highly motile in liquid cultures due to the presence of a single polar, sheathed flagellum (9). The successful invasion of B. bacteriovorus requires that it adheres to the prey cell using its type IV pilus (10, 11), which is located at the pole opposite the flagellum. Thus, the predator cell has an asymmetry that resembles the polarity of Caulobacter crescentus cells (12). During the reproductive phase of B. bacteriovorus, the prey cell dies and is transformed into a spherical structure called a bdelloplast, and the predatory cell elongates inside the bdelloplast, forming a filament. At the end of the reproductive phase, this filament undergoes synchronous septation, and progeny cells are released into the environment (13). Newly formed B. bacteriovorus cells escaped from a bdelloplast go through a maturation phase where the cell length increases (13).

Although the cell biology of B. bacteriovorus has been intensively studied (13–15), we know very little about the dynamics of chromosome replication in this predatory bacterium. Considering its small size, B. bacteriovorus possesses a relatively large chromosome (3.8 Mb) (14, 16), suggesting that it has to be efficiently compacted. The predatory chromosome contains all essential genes (e.g., those encoding the Dna proteins) and elements (e.g., an origin of chromosomal replication, *oriC*) required for its own replication. Genomic analysis also revealed the presence of structural maintenance of chromosomes (SMC) protein and a ParABS system (16), which are required in other bacteria for chromosome segregation into daughter cells (17). RNA transcriptome (RNA-seq) analysis showed that the chromosome replication-related genes of B. bacteriovorus are upregulated during the reproductive phase and downregulated during the nongrowing attack phase (18). Thus, the chromosome replication of B. bacteriovorus must be precisely coordinated with its unusual life cycle. It seems reasonable to assume that, as in other bacteria, the process is mainly regulated at the initiation step, which is a crucial cell cycle checkpoint. We recently characterized the key elements involved in the initiation of chromosome replication in B. bacteriovorus (19). We demonstrated that, as in other bacteria, B. bacteriovorus chromosome replication starts at the *oriC* region. We showed that the replication initiator protein, DnaA, from B. bacteriovorus specifically binds and unwinds its own *oriC in vitro* and *in vivo* (19). Beyond this, however, regulation of replication and the dynamics of this process during the B. bacteriovorus cell cycle are still unknown.

In recent years, the development of live cell imaging techniques has allowed direct observation of replication machinery (i.e., the replisomes) in single bacterial cells in real time. Replisomes are visualized primarily by the fusion of different replication machinery subunits to a variety of fluorescent proteins (FPs) (20–24). The FP-tagged DnaN (the β-sliding clamp) is the most widely used fluorescent fusion protein to visualize replisomes in bacteria (25); the appearance and disappearance of DnaN-FP foci indicate assembly and disassembly of the replisome complex and are considered to correspond to the initiation and termination of DNA replication, respectively.

Extensive microscopic studies have revealed that the positioning of replisomes (i.e., the replication machinery) and their dynamics during the cell cycle differ among bacteria. In some bacteria (Bacillus subtilis, Escherichia coli, and Pseudomonas aeruginosa), the replisomes are assembled in the middle of the cell, whereas in others (Caulobacter crescentus, Helicobacter pylori, and chromosome I of Vibrio cholerae) this assembly occurs at one of the cell poles. During the replication cycle, the sister replisomes may stay together at the initiation site (B. subtilis and P. aeruginosa) or travel together to the midcell (C. crescentus and H. pylori) (26–33), while in E. coli, the sister replisomes move toward the cell poles and merge again at the end of replication (30). Recent work has shown that replisome dynamics may exhibit other patterns, such as those seen for Mycobacterium smegmatis and Myxococcus xanthus (20, 34), suggesting that bacteria evolve different replication fork passage strategies that are coupled to their specific life cycle requirements.

In this study, we addressed how the dynamics of chromosome replication are coordinated with the life cycle of B. bacteriovorus. We investigated the subcellular localization of the replisome(s) in real time in single cells/filaments of B. bacteriovorus. Our data provide evidence that B. bacteriovorus exhibits a novel spatial arrangement of chromosome replication. The process starts at the invasive pole of the predatory bacterium, inside the bdelloplast, and replication proceeds until several copies of the entire chromosome are completely synthesized. This chromosome replication is not associated with cell division, and it is terminated before synchronous predator-filament septation. In addition, we observed (albeit rarely) that some B. bacteriovorus cells do not follow a canonical life cycle but rather employ two prey cell invasions to complete their life cycle if the first predation event is abortive.

## RESULTS

### Replisomes are formed during the reproductive phase.

**FIG 1:**
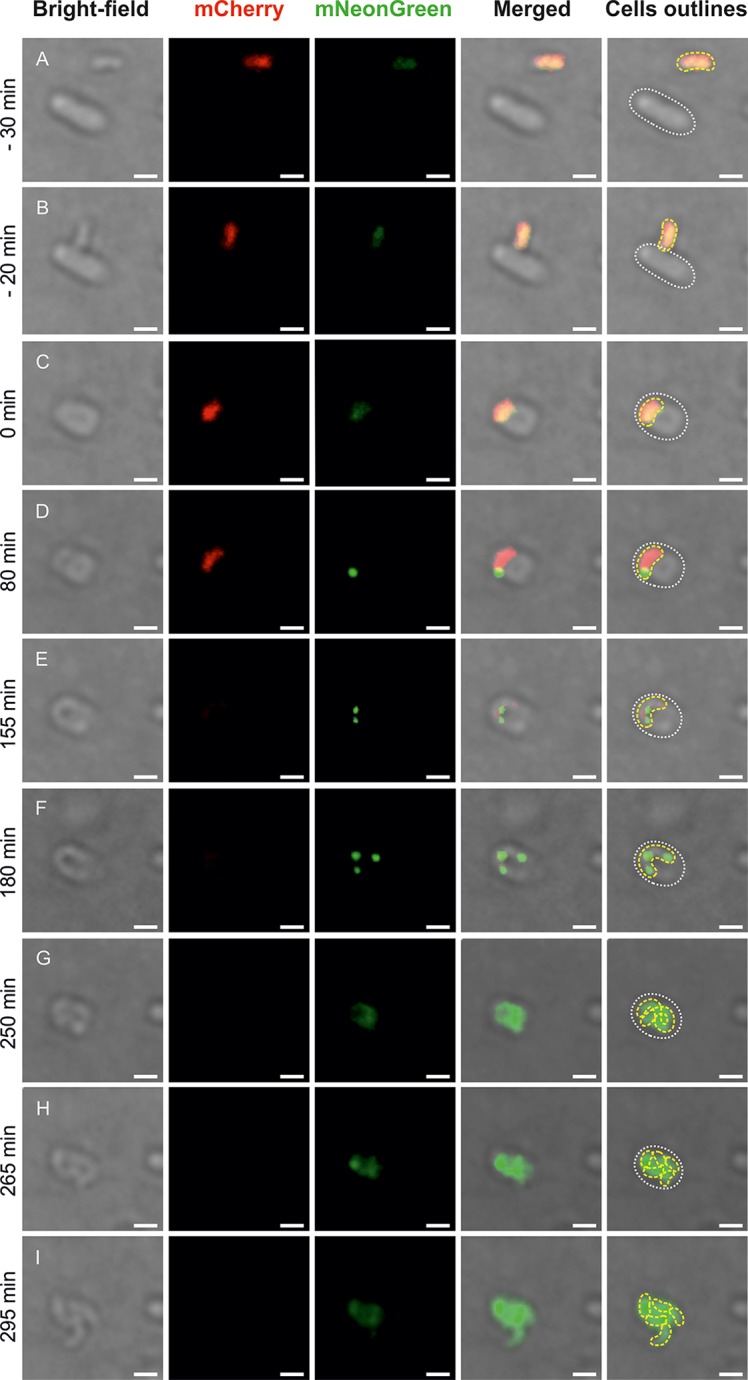
Spatiotemporal analysis of chromosome replication in a B. bacteriovorus cell growing in a bdelloplast. Time-lapse analysis of representative B. bacteriovorus cell showing the localization of replisomes (green) in a predatory cell (red) growing inside the E. coli bdelloplast. (A) Free-living predatory and host cell. (B) Attachment of B. bacteriovorus to an E. coli cell. (C) Bdelloplast formation, time = 0 min. (D) Appearance of the first replisome focus at pilus pole (see Fig. 2) of B. bacteriovorus cell—the start of chromosome replication. (E and F) Further growth and chromosome replication. (G) Termination of predatory chromosome replication. (H) The beginning of B. bacteriovorus filament septation. (E) The release of progeny cells from the bdelloplast. Red indicates PilZ-mCherry-labeled cytoplasm of attack-phase B. bacteriovorus cells, and green indicates DnaN-mNeonGreen of B. bacteriovorus. Photos represent merged bright-field and fluorescence (red and green) images. The B. bacteriovorus cell and the bdelloplast are marked by yellow and white dotted lines, respectively. Bar, 1 μm.

To monitor the positioning of the replisome in B. bacteriovorus cells, we constructed strain HD100 DnaN-mNeonGreen/PilZ-mCherry, which produced the β-sliding clamp (DnaN) in fusion with mNeonGreen protein in the *pilZ-mCherry* background (Fig. S1A; see also Materials and Methods). PilZ (Bd0064; a protein that binds cyclic di-GMP) is localized nearly constitutively (35) throughout the cytoplasm of the attack-phase B. bacteriovorus cell, so its fluorescent tagging allowed us to label the entire predatory cell in red during the attack phase and in the early stage of the reproductive phase (Fig. 1A to D and Fig. S1B). The DnaN-mNeonGreen/PilZ-mCherry strain exhibited a predatory kill curve, duration of reproductive phase, and predation efficiency similar to those of the wild-type strain (Fig. S2), suggesting that the fusion proteins were fully functional.

To analyze the duration and timing of B. bacteriovorus chromosome replication, we used an agarose pad in combination with ibidi cell-imaging dishes (see Materials and Methods). In this system, the predatory cells could move freely beneath the agarose pad, whereas the immobilized prey cells (i.e., E. coli) were able to form bdelloplasts. We were thus able to observe the complete life cycle of B. bacteriovorus. Microscopic analysis revealed that DnaN-mNeonGreen fluorescence was constantly present in predatory cells, either as a dispersed signal found throughout the cell during the (nonreplicating) attack phase and shortly before septation or as discrete diffraction-limited foci observed throughout the most of the reproductive phase (inside the bdelloplast) (Fig. 1). From this, we infer that the diffuse fluorescence and the fluorescent foci reflect disassembled (or not yet assembled) replisomes and ongoing chromosome replication, respectively. In the reproductive phase, we observed up to four DnaN-mNeonGreen foci per single B. bacteriovorus filament. Most of the cells growing inside the bdelloplast contained two (28%), three (46%), or four (25%) visible replisomes (Table 1). Only a small fraction of cells (1%) contained more than four replisomes. As expected, longer filaments of B. bacteriovorus usually contained more replisomes than shorter ones.

**TABLE 1:** Visible replisomes in growing B. bacteriovorus cells

No. of visible replisomes	No. of cells	Fraction (%)
2	31	28
3	51	46
4	28	25
>4	1	1

To examine whether the observed appearance of fluorescent foci legitimately reflected ongoing replication, we performed an experiment in which novobiocin was added to the agarose pad (200 μg/ml). This agent inhibits DNA replication and thus replisome assembly by acting on DNA gyrase, which normally, through relaxation of positive supercoils ahead of the replication fork, resolves the torsional tension and allows DNA synthesis progression (36). When predatory and host cells were added to the agarose pad with novobiocin, bdelloplasts were formed, although the fluorescence foci did not appear (only diffuse fluorescence was seen) (Fig. S1C). This confirms that the DnaN-mNeonGreen foci represented active replisomes.

### Chromosome replication starts at the invasive pole of the B. bacteriovorus cell, and two or more replisomes are usually observed in a single filament.

**FIG 2:**
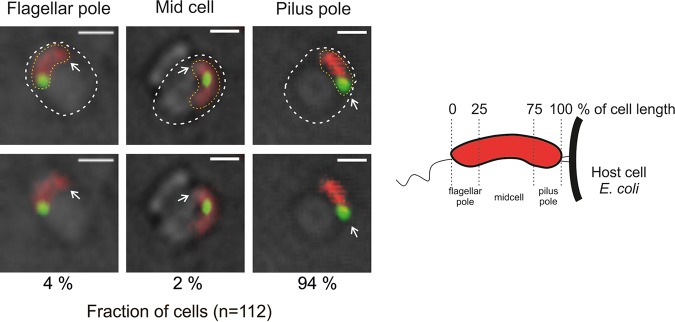
Localization of the first replisome in relation to the pilus pole of B. bacteriovorus. B. bacteriovorus cells (red) with replisome (green) localized in the vicinity of a pole (flagellar or pilus) or in the midcell are shown. The white arrow indicates the pilus pole, determined by watching predator entry, which is pilus first. A schematic of a B. bacteriovorus cell is depicted on the right. The cell is divided into subregions according to the percentage of cell length. Images were recorded every 60 s. Red indicates PilZ-mCherry-labeled cytoplasm of attack-phase B. bacteriovorus cells, and green indicates DnaN-mNeonGreen of B. bacteriovorus. All photos represent merged bright-field and fluorescence (red and green) images. The B. bacteriovorus and E. coli cells are marked by yellow and white dotted lines, respectively. Bar, 1 μm.

A B. bacteriovorus cell enters a prey cell by using the type IV pili located on the nonflagellate pole of the predatory bacterium (11, 37). Careful tracking of predatory entry into E. coli cells allowed us to observe the appearance of the first focus (i.e., replisome) in relation to the given cell pole of B. bacteriovorus. The images of predatory cells were acquired every 60 s using time-lapse fluorescence microscopy (TLFM). The TLFM analysis showed that all analyzed B. bacteriovorus cells after entering E. coli did not flip inside the prey’s periplasm (see Fig. S3 and Movie S1). In 94% of cells (*n* = 111, Fig. 2), the first replisome was assembled at the invasive (pilus-proximal) pole of the cell. In a small fraction of cells (6%), the first replisome was observed either at the flagellar pole or at the midcell (Fig. 2). Microscopic investigations revealed that the first fluorescent focus appeared at 96 ± 29 min (*n* = 111) after the attachment of B. bacteriovorus to the E. coli cell and at 74 ± 26 min (*n* = 111) after bdelloplast formation. The time intervals between the appearances of consecutive replisomes varied (Table S1). The second fluorescent focus was assembled 59 ± 20 min (*n* = 111) after appearance of the first, while the third and fourth replisomes appeared (when relevant) after shorter time intervals of 32 ± 18 min (*n* = 80) and 27 ± 15 min (*n* = 28), respectively (Table S1). Replisome positions (except for that of the first replisome, see below) were not restricted to specific cell regions. Although the B. bacteriovorus could be visualized inside the bdelloplast, it was difficult to determine the positions of replisomes within a growing filament because they were highly mobile and frequently mixed with each other (Movie S2). Moreover, during the late stage of B. bacteriovorus cell growth, the filament can reach a length exceeding the bdelloplast diameter and begin to curve and overlap itself (13, 15, 38).

In summary, our results indicate that B. bacteriovorus chromosome replication is initiated at the formerly piliated invasion predator pole and that multiple replisomes are highly dynamic during the reproductive phase.

### The number of progeny cells is proportional to the duration of chromosome replication.

**FIG 3:**
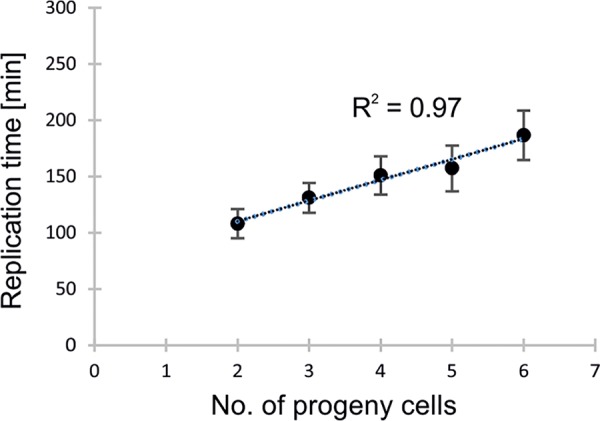
Correlation between the number of progeny cells and the duration of chromosome replication. Correlation coefficient (*R*^2^) = 0.97, *n* = 112.

To determine the duration of DNA replication (C period) during the reproductive phase of B. bacteriovorus, we measured the time from the appearance of the first focus/replisome (regarded as initiation) to the disappearance of the last focus/replisome (estimated to be termination) (Fig. 1). We assumed that DNA synthesis starts with no delays after and/or before replisome assembly. The average duration of chromosome replication was 144 ± 26 min (range, 112 to 187 min; *n* = 112; Fig. 3 and Table 2). Because the length of the C period varied significantly between bdelloplasts, we analyzed the relationship between the number of progeny cells released from the bdelloplasts and the duration of DNA replication (B. bacteriovorus growing in abnormally elongated host cells, see below, was excluded from the regression analysis). As expected, the number of progeny cells was positively correlated with the duration of B. bacteriovorus chromosome replication (correlation coefficient [*R*^2^] = 0.97; Fig. 3). In all cells, the replication process was terminated up to 21 min (*n* = 98) prior to filament septation (D period).

**TABLE 2:** Duration of chromosome replication in different progeny cells

No. of progeny cells	Free-living cells		Newly released cells	
	Duration of replication (min)	SD	Duration of replication (min)	SD
2	112	16		
3	128	20	122	11
4	151	17	140	14
5	158	19	147	23
6	187	22	170	7

During our TLFM analyses, we observed that E. coli occasionally formed extremely elongated cells (Fig. S4A) and that B. bacteriovorus easily invaded these cells and formed a huge, oval bdelloplast (Fig. S4B). As noted by Kessel and Shilo (38), we observed that the B. bacteriovorus filament reached an abnormal length in such bdelloplasts, presumably due to greater nutrient supply. The elongated filament contained numerous replisomes (up to six; see Fig. S4E) that were evenly positioned within the growing predatory filament and appeared sequentially (Movie S3). In such cases, more progeny cells were released from abnormal bdelloplasts (Fig. S4F and Movie S3).

### Replication begins earlier in progeny predator cells that immediately invade new prey cells than in free-living predatory cells that invade prey cells.

After being released from the bdelloplast, a progeny cell might attack prey in the surrounding neighborhood or actively move to more distantly located prey, taking several minutes to do so. We observed that if newly released progeny cells were in the close vicinity of another prey cell, they could quickly attack these prey (within 10 ± 5 min; *n* = 39) and form a new bdelloplast. In such bdelloplasts, replication started significantly earlier (23 ± 11 min) than in the case of bdelloplasts formed by free-living mature predatory cells (74 ± 26 min) (*P* value < 0.001, *n* = 39; Table S1), but we did not notice any significant differences in the duration of replication between newly released predatory cells (140 ± 20 min) and free-living cells (144 ± 26 min) (*P* > 0.05). Moreover, as in mature, free-living B. bacteriovorus cells (Table 2), in newly released cells, the length of the C period was positively correlated with the number of progeny cells (data not shown).

These findings indicate that newly released progeny cells that rapidly invade new prey cells show an earlier initiation of chromosome replication than free-living B. bacteriovorus cells that invade prey cells, suggesting a time course of resetting to nonreplicative attack phase after prey exit.

### Some B. bacteriovorus cells might require two prey cells to complete their life cycle.

**FIG 4:**
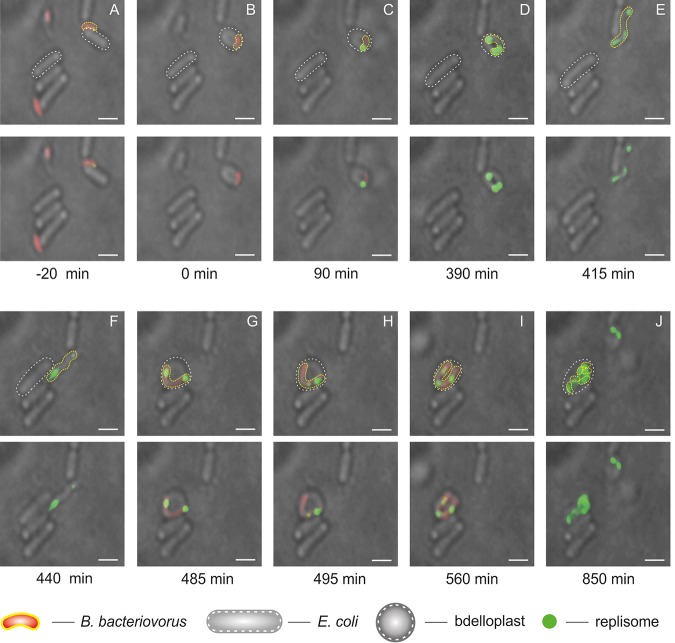
A rare example of a B. bacteriovorus cell life cycle conducted in two independent E. coli host cells. (A) B. bacteriovorus attachment to E. coli. (B) Bdelloplast formation, time = 0 min. (C and D) Growth and replication in the first host cell. (E) Novel release of the nonseptated predatory filament from the first host cell. (F) The attack of a nonseptated predatory filament on another prey cell. (G) Bdelloplast formation. (H and I) Growth and replication in the second host. (J) Filament septation inside the bdelloplast. Red indicates PilZ-mCherry-labeled cytoplasm of B. bacteriovorus attack-phase cell, and green indicates DnaN-mNeonGreen of B. bacteriovorus. Photos represent merged bright-field and fluorescence (red and green) images. The B. bacteriovorus and E. coli cells are marked by yellow and white dotted lines, respectively. Bar, 1 μm.

Careful TLFM analysis of B. bacteriovorus cells allowed us to observe a predator cell that did not complete its life cycle within the E. coli bdelloplast. Consistent with results published by Fenton et al. (13), we noticed that the filament failed to undergo septation. The undivided filament escaped the bdelloplast (whether actively or passively is the subject of further research beyond this paper) and encountered another prey cell, within which it completed its life cycle (Fig. 4). In such a “two-stage” growing phase, the predator cell replicated its chromosome inside the first bdelloplast but retained visible replisomes for a much longer period than that seen in the normal life cycle (390 versus 187 min; Fig. 4D). The replisome was observed in the filament even after its release from the first prey cell (Fig. 4E and Movie S4). Upon its release, this elongated B. bacteriovorus filament entered a new prey cell, whereupon chromosome replication proceeded (Fig. 4H and I and Movie S4). Growth in a second prey cell ended with filament septation (Fig. 4J).

These observations indicate that, rarely, a B. bacteriovorus cell might require two independent prey cells to complete its life cycle.

## DISCUSSION

**FIG 5:**
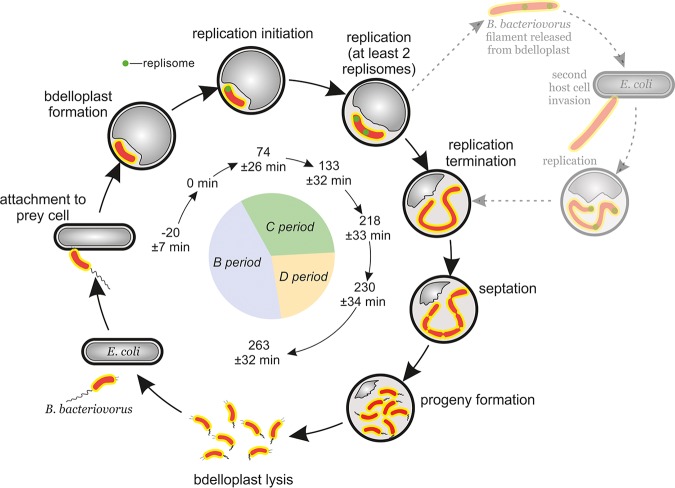
Dynamics of chromosome replication during the B. bacteriovorus life cycle. B. bacteriovorus (orange) attacks and invades the host cell (gray). Chromosome replication (green replisome) is initiated at the invasive pilus-proximal pole. The dotted line and greyscale represent the speculated alternative B. bacteriovorus life cycle conducted in two independent host cells. The inner circle diagram represents the periods of the bacterial cell cycle as follows: B, the time between progeny formation and the initiation of chromosome replication in daughter cells; C, chromosome replication; and D, the time between the termination of replication and the completion of filament septation. *t* = 0 min refers to the bdelloplast formation. The listed time points were calculated in this study.

The chromosome replication of B. bacteriovorus occurs only during the reproductive phase within the prey while the motile, free-living cells are incapable of initiating chromosome replication. Thus, as in C. crescentus, the chromosome replication process must be strictly regulated and coordinated with the unusual life cycle of this predatory bacterium (3, 38) (Fig. 5). Although chromosome replication dynamics have been relatively well studied in several species of Gram-positive and Gram-negative bacteria, almost nothing is known about this process in predatory bacteria. To address this, we developed a TLFM-based system that allowed us to observe chromosome replication dynamics in a single cell of B. bacteriovorus growing inside the prey bacterium, E. coli. Here, we report that this predatory bacterium exhibits a novel spatiotemporal arrangement of chromosome replication dynamics. Moreover, we found that B. bacteriovorus cells are capable of using two independent prey cells to complete their life cycle if the first predation event fails.

Our data indicate that the chromosome replication of B. bacteriovorus starts at the invasive pole (Fig. 2). This pole is essential for predation, especially for the entry of this bacterium into its prey (11). Moreover, a regulatory protein hub controlling predatory invasion was discovered at this pole (39). The pili protruding from the invasive pole take part in sequentially sensing the stepwise phase transition in B. bacteriovorus (39, 40). During prey recognition, the pili mediate the transduction of a yet-unidentified early signal that occurs in the cytoplasmic membrane of the host (39). The second cue, which also has not yet been specified, originates from the prey cytoplasm and is believed to promote DNA replication (40). Thus, the invasive pole of B. bacteriovorus seems to be involved in the transition from the attack phase to the reproductive phase. We speculate that during the transition phase, chromosome replication is triggered by a yet-unknown regulator(s), presumably by a signal transduction cascade(s), and that this process is likely to be mediated by the recognition of the cue arising from the invasive pole (39, 40).

Unlike B. bacteriovorus, model bacteria such as E. coli and B. subtilis undergo replisome assembly in the middle of the cell (28, 30, 31). Interestingly, C. crescentus and V. cholerae (chromosome I) resemble B. bacteriovorus both in their asymmetry and in assembling their replisomes at a cell pole (27, 41). In C. crescentus and V. cholerae (chromosome I), the subcellular localization of *oriC* (and thus the sites of replisome assembly) is determined by the specific *oriC*-anchoring proteins PopZ and HubP, respectively (42, 43). None of the genes of B. bacteriovorus encode a protein that is homologous to PopZ or HubP. Thus, the factor(s) responsible for anchoring the B. bacteriovorus *oriC* region at the invasive pole remains to be identified.

Spatiotemporal analysis of the chromosome dynamics in B. bacteriovorus revealed that the first replisome appears 96 ± 29 min and 74 ± 26 min after the attachment of the predatory cell to the prey cell and the formation of the bdelloplast (Fig. 1), respectively. This pronounced delay in the initiation of chromosome replication (replisome assembly) presumably reflects the unusual predatory behavior of B. bacteriovorus. After entering a prey cell, the predatory cell must adapt to growth in the bdelloplast before it can begin DNA replication. Indeed, metaanalyses of gene expression profiles (RNA-seq and microarray profiling) have demonstrated that during the first 60 min postinfection, genes involved in growth and replication are highly upregulated (18, 44). Our present results show that DNA is not yet being synthesized at this time (Fig. 1). Thus, we speculate that as-yet-undiscovered replication checkpoints act to coordinate the cell cycle progression and DNA replication of B. bacteriovorus. The predatory cell modifies the structure of the host’s peptidoglycans to make the environment more flexible and suitable for filamentous growth (45–50). Moreover, during the adaptation inside the bdelloplast, B. bacteriovorus releases hydrolytic enzymes to the prey’s cytoplasm to degrade various prey macromolecules and uses these components to build its own cellular structures (18). The chromosome replication of B. bacteriovorus is assumed to be triggered only after the bacterium adapts to the growth conditions inside the bdelloplast.

Using bdelloplasts that produced only two progenitor cells (11%; *n* = 112), we were able to calculate the rate of DNA synthesis. Given the length of the C period for such cells (112 min), the rate of DNA synthesis is about 300 nucleotides (nt)/s. This is ∼2 to 3 times slower than that of E. coli (600 to 1,000 nt/s [51]). The activity of B. bacteriovorus DNA polymerase III is not likely to be the rate-limiting factor, since subunits of the holoenzyme show high homology with the corresponding subunits from E. coli (crucial amino acids for catalytic activity of the α subunit of DNA polymerase III are identical in B. bacteriovorus and E. coli; data not shown). Thus, the DNA synthesis rate of B. bacteriovorus is presumably limited by the availability of nutrients, particularly nucleotides (see below).

In filaments that formed more than two progeny cells, the C period ranged up to 187 min, indicating that in these filaments, reinitiation of chromosome replication must take place; to synthesize three or more chromosomes within less than 187 min, a new round of replication must be initiated before the previous round is completed. Thus, the reinitiation mechanism ensures that each of the nascent progeny cells receives a single chromosome.

The duration of the reproductive phase in B. bacteriovorus, including the C period, varies between cells, but it is not yet known how the length of this phase is regulated. Gray and Ruby (52) suggested that a prey-derived regulatory factor(s) may be involved in the developmental cycle of B. bacteriovorus, operating at the level of the cell’s decision to either continue or terminate the reproductive phase. As in other bacteria, B. bacteriovorus presumably adjusts its size and growth rate according to the availability of nutrients. Indeed, a B. bacteriovorus cell that attacks a large (i.e., large nutrient pool) prey cell will synthesize more chromosomes and develop a longer filament (38; see also Fig. S4) and thus release more progeny cells (Fig. S4F). To synthesize 2 to 3 nascent chromosomes, the predatory cell utilizes the DNA and RNA of prey as direct sources of nucleotides (53, 54), but synthesis of more chromosomes (and consequently progeny cells) requires *de novo* synthesis of nucleotides from carbon and nitrogen precursors, including amino acids obtained by hydrolysis of the prey’s proteins (54). Nevertheless, we cannot exclude the possibility that the highly compacted B. bacteriovorus chromosome slows down the replication fork movement.

Surprisingly, we observed some B. bacteriovorus cells in which replication was initiated relatively shortly after their invasion into new prey cells. This occurred only among newborn predatory cells that were released in close proximity to new prey cells, invading them immediately upon release. In such predatory cells, replication began significantly earlier than that in free-living predatory cells that underwent invasion (23 min versus 74 min, respectively; *P* < 0.001; Table S1). It can be assumed that the proteins involved in chromosome replication (e.g., the initiator protein, DnaA) are not completely degraded in these early-replicating B. bacteriovorus cells. In C. crescentus, which also exhibits a biphasic life cycle, DnaA (DnaA*_Cs_*) undergoes cell cycle-controlled proteolysis mediated by the Lon protease (55, 56). The accumulation of DnaA*_Cs_* in replication-active cells of C. crescentus corresponds to a low synthesis level of CtrA, which represses chromosome replication initiation (55, 56). Controlled proteolysis of DnaA and/or repression of chromosome replication by a CtrA-like protein could possibly occur in B. bacteriovorus during the attack phase. In this scenario, the level of this putative replication repressor might be too low to inhibit replication in newly released cells, and additionally, such cells could contain levels of replication proteins sufficient to restart chromosome replication.

In B. bacteriovorus, chromosome replication is not immediately followed by cell division; instead, a multinucleoid filament is formed. Such replication dynamics resembles that found in the vegetative and aerial mycelia of Streptomyces species (57, 58). Moreover, after termination of replication, the multinucleoid filament (similar to the sporulating aerial hyphae of *Streptomyces*) undergoes synchronous septation (up to 21 min after replication termination) to ensure that each nascent predatory cell receives a single copy of the chromosome. Thus, in contrast to the model organisms (E. coli and B. subtilis), B. bacteriovorus exhibits extended B and D periods; the chromosome replication begins approximately 74 min after bdelloplast formation and is terminated before filament fragmentation inside the bdelloplast.

To conclude, we show here that the predatory cells of B. bacteriovorus exhibit an unusual spatiotemporal arrangement of chromosome replication dynamics that combine different features from Gram-negative and Gram-positive bacteria. The chromosome replication of B. bacteriovorus initiates at a specific cell pole (the invasion one), as also seen in other asymmetrical bacteria, C. crescentus and V. cholerae (chromosome I). Interestingly, we observed “cell-to-cell” variation in the replication dynamics. In a “rich” environment, i.e., in a dense prey cell population, the newly released, not fully matured predatory cells are able to quickly attack prey in the surrounding neighborhood and begin the chromosome replications earlier (see Table S1). In larger prey cells that provide more nutrients, B. bacteriovorus grows as a long filament that exhibits high replication activity, resulting in the synthesis of more chromosomes (up to 12). On the other hand, in the case where B. bacteriovorus predation is abortive (e.g., due to the small size of prey; Fig. 4), the predatory bacterium can complete its chromosome replication and consequently its cell cycle by encountering and invading another prey cell (Fig. 5). We speculate that heterogeneity in replication dynamics may reflect a relaxation of cell cycle checkpoints, possibly increasing the ability of predatory cells to adapt to the specific conditions of different prey—remembering that these predators replicate within a wide range of different prey genera. Thus, the population of B. bacteriovorus, like other bacterial populations, is not homogenous, and some individuals can show unique features different from others.

## MATERIALS AND METHODS

### DNA manipulations, bacterial strains, and culture conditions.

DNA manipulations in E. coli were carried out using standard protocols (59). Reagents and enzymes were supplied by Thermo Scientific and Sigma-Aldrich. Oligonucleotides were synthesized by Sigma-Aldrich. The plasmids used to construct B. bacteriovorus HD100 strain DnaN-mNeonGreen/PilZ-mCherry (see below) were propagated in E. coli DH5α, grown in LB broth or on LB agar plates (supplemented with 50 μg/ml kanamycin), and then transformed into E. coli S17-1. The latter were grown in liquid culture in YT medium (0.8% Bacto tryptone, 0.5% yeast extract, and 0.5% NaCl [pH 7.5]) with (S17-1 pZMR100) or without (S17-1) kanamycin (50 μg/ml), at 37°C with shaking (180 rpm). B. bacteriovorus was grown by predation on E. coli S17-1 or E. coli S17-1 pZMR100 (kanamycin-resistant strains) in Ca-HEPES buffer (25 mM HEPES and 2 mM calcium chloride [pH 7.6]), as described in Lambert et al. (60). Details regarding the utilized strains, plasmids and oligonucleotides are listed in Table 3.

**TABLE 3:** Bacterial strains, primers, and plasmids^*a*^

Bacterial species, strain, primer, or plasmid	Description/sequence	Reference or source
Species and strains		
*E. coli*		
S17-1	*thi pro hsdR^−^ hsdM^+^ recA*; harboring plasmid RP4-Tc::Mu-Kn::Tn*7*, used as donor for conjugation of plasmids into *Bdellovibrio*	61
S17-1 pZMR100	S17-1 strain containing pZMR100 plasmid to confer Kan^r^; used as Kan^r^ prey for *Bdellovibrio*	62
*B. bacteriovorus*		
HD100*Bd0064-mCherry*	HD100 with replaced *bd0064(pilZ)* gene to *bd0064-mCherry* in the native locus	35
HD100*Bd0064-mCherry*/*Bd0002-mNeon*	HD100*Bd0064-mCherry* single crossing-over strain carrying integrated plasmid pK18_mNeon_dnaN at the *dnaN* (Bd0002) locus	This work
Primers		
pK18_dnaN(Gib)F	CGTTGTAAAACGACGGCCAGTGCCAATGAAATTAGAGATTGATAAGCG	
mNeon_dnaN(Gib)R	CTTTCGAAACCATGATTCTCATTGGCATCAC	
dnaN_mNeon(Gib)F	GCCAATGAGAATCATGGTTTCGAAAGGAGAG	
pK18_mNeon(Gib)R	GGAAACAGCTATGACCATGATTACGTCACTTATAGAGTTCATCCATACC	
Plasmids		
pAKF220	Plasmid carrying *mNeonGreen* coding sequence; Amp^r^	Andrew K. Fenton
pK18*mobsacB*	Suicide vector used for conjugation and recombination into *Bdellovibrio* genome; Kan^r^	63
pK18_dnaN_mNeon	Derivative of pK18*mobsacB* containing fusion gene *bd0002(dnaN)-mNeonGreen*; Kan^r^	This work

a Kan^r^, kanamycin resistant; Amp^r^, ampicillin resistant.

### Construction of B. bacteriovorus strain HD100 DnaN-mNeonGreen/PilZ-mCherry.

We constructed B. bacteriovorus strain HD100 DnaN-mNeonGreen/PilZ-mCherry, in which the cytoplasm was labeled red by the PilZ fusion and the replisome labeled green by the DnaN fusion. We amplified the coding sequences of *dnaN* [primers pK18_dnaN(Gib)F and mNeon_dnaN(Gib)R] and *mNeonGreen* [primers dnaN_mNeon(Gib)F and pK18_mNeon(Gib)R] using chromosomal B. bacteriovorus HD100 and pAKF220 (plasmid kindly provided by Andrew K. Fenton), respectively, as the templates. Gibson assembly was used to clone the PCR products into pK18*mobsacB*. The obtained construct (pK18*dnaN-mNeonGreen*) was transformed into E. coli S17-1 and conjugated to B. bacteriovorus strain HD100 PilZ-mCherry as described previously (60). Single crossing-over of pK18*dnaN-mNeonGreen* into the B. bacteriovorus chromosome replaced the wild-type copy of *dnaN* with the DnaN-mNeonGreen fusion-encoding gene (Fig. S1A). From this, we obtained a B. bacteriovorus strain with the *dnaN-mNeonGreen* fusion under the control of the endogenous promoter and a second disrupted and nonexpressed copy of the *dnaN* gene. Proper construction of the DnaN-mNeonGreen/PilZ-mCherry strain was verified by PCR, sequencing, and Western blotting.

### Time-lapse fluorescence microscopy.

Cells of B. bacteriovorus strain DnaN-mNeonGreen/PilZ-mCherry were prepared by predation on E. coli S17-1 pZMR100 in 50 ml Ca-HEPES buffer in the presence of 50 μg/ml kanamycin. The culture was spun down at 5,500 rpm for 20 min at 30°C, resuspended in 5 ml of Ca-HEPES buffer, and incubated at 30°C with 200 rpm shaking for 30 min. Agarose gel (1%) in Ca-HEPES buffer with or without novobiocin (final concentration, 200 μg/ml) was poured into a 35-mm glass-bottom μ-Dish (ibidi) and allowed to solidify. The gel was removed from the dish, flipped over to bottom-up and coated with E. coli S17-1 overnight culture. Next, a few drops of B. bacteriovorus suspension were added on the E. coli-coated surface and spread by inoculation loop. Agarose gel prepared in this way was placed back in a 35-mm glass-bottom μ-Dish bottom-down. Images were recorded every 1 or 5 min using a Delta Vision Elite inverted microscope equipped with an Olympus 100×/1.40 and a Cool SNAP HQ2-ICX285 camera. PilZ-mCherry was visualized with mCherry (EX575/25; EM625/45) and neutral density (ND; 50%) filters with an exposure time of 200 ms. DnaN-mNeonGreen was visualized with green fluorescent protein (GFP) (EX475/28; EM525/48) and ND (50%) filters, with an exposure time of 80 ms. Bright-field images were taken with an ND (5%) filter and exposure time of 50 ms. The captured images were analyzed using the ImageJ Fiji suite (http://fiji.sc/Fiji).

TLFM experiments were done in three independent biological replicates.

## Supplementary Material

Supplemental file 1

Supplemental file 2

Supplemental file 3

Supplemental file 4

Supplemental file 5
