# Supplementary material for: Dynamics of Chromosome Replication and Its Relationship to Predatory Attack Lifestyles in Bdellovibrio bacteriovorus
Source: Appl Environ Microbiol. 2019 Jul 1;85(14):e00730-19. doi: 10.1128/AEM.00730-19 (PMC6606864; doi:10.1128/AEM.00730-19)
Supplement: Supplemental file 1 [file AEM.00730-19-s0001.pdf]

Supplemental Materials

Supplemental Materials and Methods

Prey survival assay of *B. bacteriovorus* strains

Cells of *B. bacteriovorus* strains DnaN-mNeonGreen/PilZ-mCherry and wild-type were prepared by predation on *E. coli* S17-1 in 50 ml Ca-HEPES buffer. The cultures were filtered through 0.45 µm filters, spun down at 6000 rpm for 20 min at 30°C and resuspended in 510 µl of Ca-HEPES buffer giving the final concentration of approximately $1x10^9$ pfu/ml (plaque-forming unit/ml). *E. coli* S17-1 overnight culture was spun down at 6000 rpm for 10 min at 20°C, washed with Ca-HEPES buffer and diluted to OD_{600}=1.0 with Ca-HEPES buffer giving the final concentration of approximately $1x10^9$ cfu/ml. The assay was set up by adding 500 µl of *B. bacteriovorus* cells (DnaN/PilZ or wild-type) or Ca-HEPES buffer (control) to 5 ml of *E. coli* S17-1 (OD=1.0). Cultures were incubated at 30°C with 200 rpm shaking. To enumerate predatory cells concentration taken to predation assay dilutions of *B. bacteriovorus* cells were plated on overlay agar plates. Plates were incubated at 30°C for 5 days. To enumerate colony forming units (cfu) of prey cells, samples were taken at 0h, 3h, 6h, and 24h and plated in triplicate by Misra and Miles technique on YT agar plates at serial dilutions. Plates were incubated overnight at 37°C and then, visible *E. coli* colonies were enumerated. Pray survival assay was done in two independent biological replicates.

Predatory kill curves of *B. bacteriovorus* strains

B. bacteriovorus strains (DnaN-mNeonGreen/PilZ-mCherry and wild-type) were prepared as described above. *E. coli* S17-1 overnight culture was spun down at 6000 rpm for 10 min at 20°C, washed with Ca-HEPES buffer and diluted to OD_{600} = 1.0 with Ca-HEPES buffer. 20 µl of filtrated *B. bacteriovorus* cells were added to 280 µl of *E. coli* suspension. Lysis curves were analyzed using Bioscreen C (Automated Growth Curves Analysis System, Growth Curves USA) by measuring the decrease of optical density (OD_{600}) at 30°C in 20-minute intervals for 27 hours. Experiments were done in three independent biological replicates.

Supplemental Figures

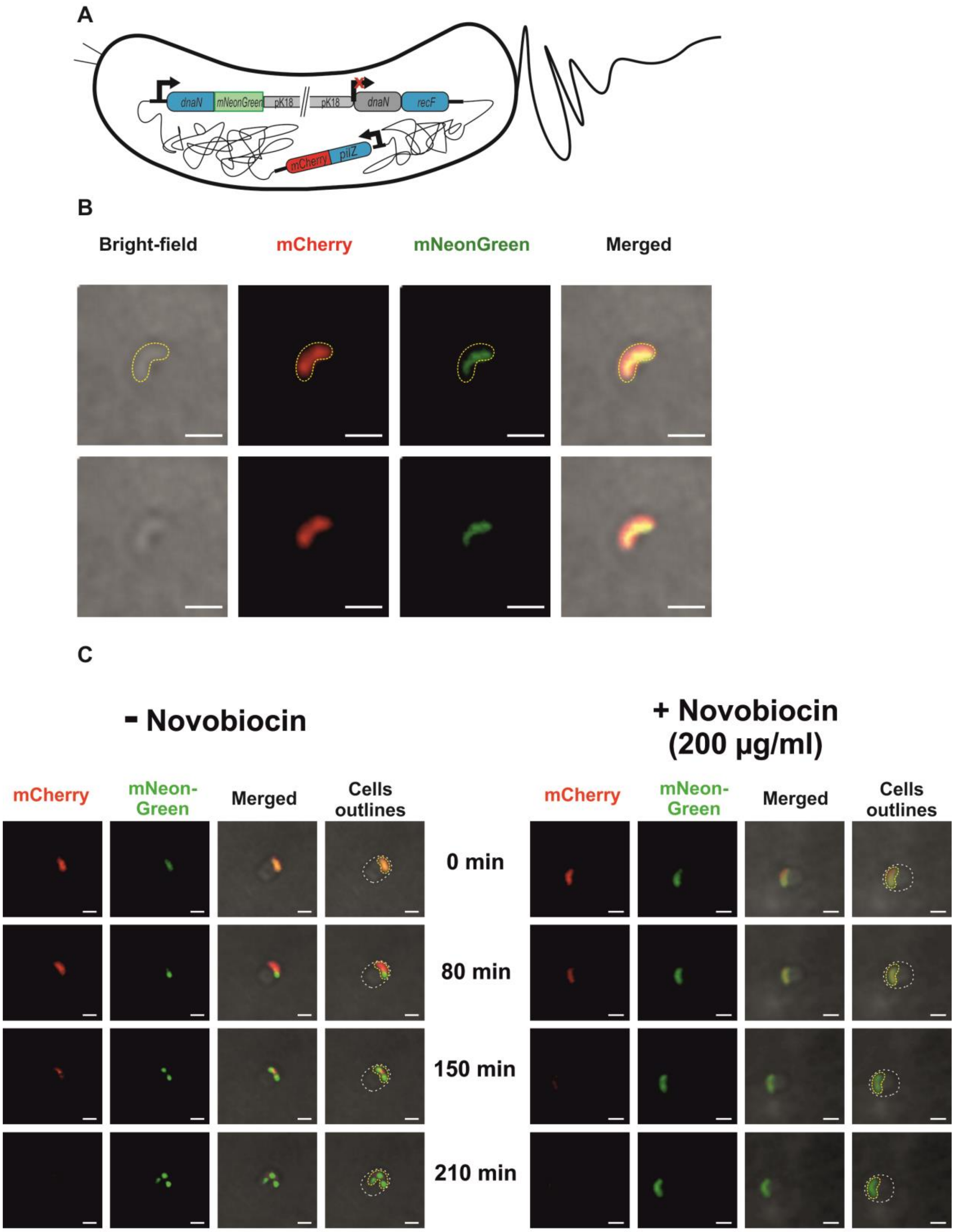

Figure S1 Characteristics of the *B. bacteriovorus* strain, DnaN-mNeonGreen/PilZ-mCherry

(A) Cartoon of the single crossover integration of pK18*dnaN-mNeonGreen* into the *B. bacteriovorus* chromosome. Black arrows indicate gene promoters. **(B)** PilZ-mCherry and DnaN-mNeonGreen in *B. bacteriovorus* attack-phase cells, as assessed under epifluorescence microscopy. The following are shown (beginning on the left): bright-field image; red fluorescence image; green fluorescence image; and merged bright-field and fluorescence images. **(C)** Effect of novobiocin on the presence of replisome foci. *B. bacteriovorus* overnight culture was collected and split into two equal parts. The predation of *B. bacteriovorus* DnaN-mNeonGreen/PilZ-mCherry on *E. coli* S17-1 cells was observed using time-lapse fluorescence microscopy on 1% agarose gel in Ca-HEPES buffer with and without novobiocin (200 µg/ml, final concentration). Left panel: time-lapse analysis of *B. bacteriovorus* replisomes without the presence of novobiocin (control). Right panel: time-lapse analysis of *B . bacteriovorus* replisomes in the presence of novobiocin. Scale bar = 1 µm.

A

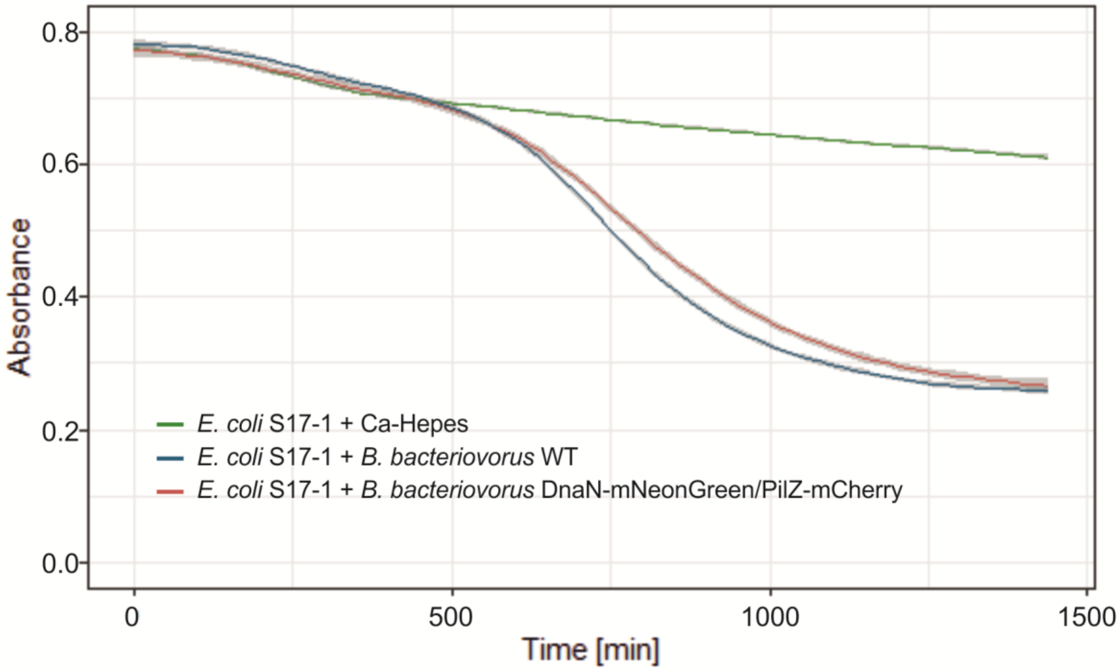

B

	WT	DnaN/PilZ	Control	
**0 h**	**9×10^8**	**9.5×10^8**	**9.5×10^8**	
**3 h**	**2.5×10^7**	**1.3×10^5**	**8.3×10^8**	***E. coli* S17-1 cells survivors**
**6 h**	**8.3×10^3**	**1×10^4**	**7.5×10^8**	
**24 h**	**4.2×10^3**	**1.2×10^3**	**6.2×10^8**	

C

	Wild-type	DnaN/PilZ
**Mean [min]**	254	263
**SD**	33	32

Figure S2 Predatory kill curve and predation efficiency of B. bacteriovorus wild-type and DnaN-mNeonGreen/PilZ-mCherry

(A) Predation kill curve of *B. bacteriovorus* strains

(B) Cfu/ml of *E. coli* cells after incubation with examining *B. bacteriovorus* strains. In red pfu/ml of *B. bacteriovorus* cells.

(C) Duration of reproductive phase determined as the time from the entering of *B. bacteriovorus* into *E. coli* to the leaving of nascent cells.

For each strain 79 prey cells invasions were analysed.

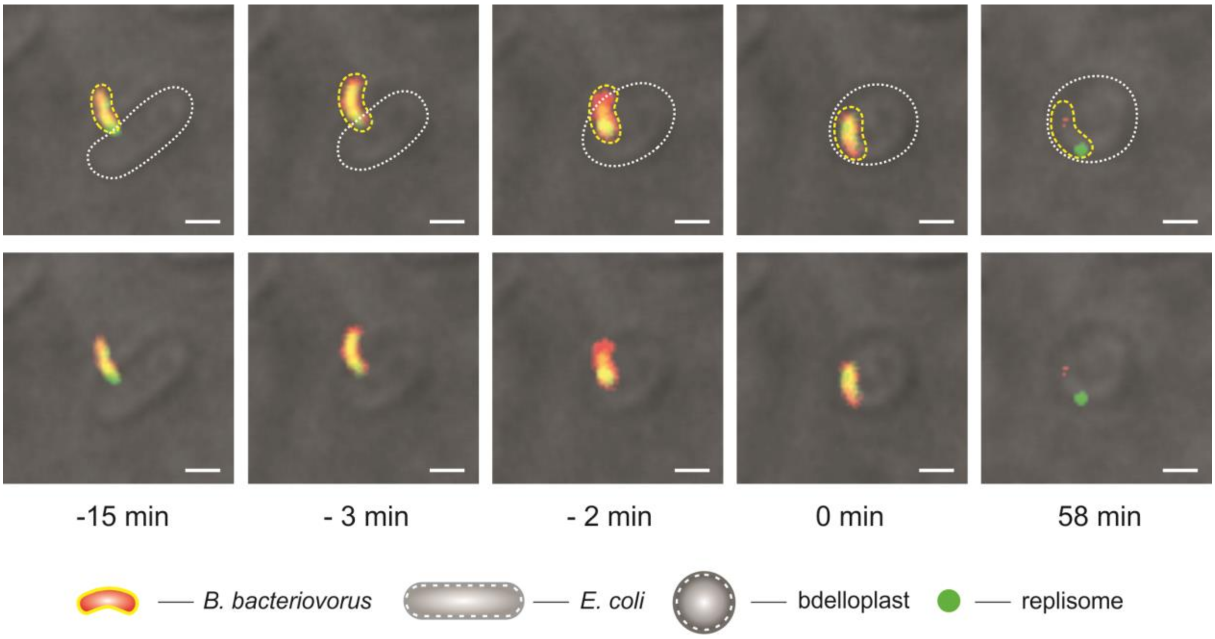

Figure S3 *B. bacteriovorus* entry and invasive pole localization in *E. coli* periplasm

Time-lapse analysis of representative *B. bacteriovorus* cell showing predatory entry to prey periplasm. (**A**) Attachment of *B. bacteriovorus* to *E. coli* host cell. (**B-D**) Predatory entry into prey's periplasm in relation to the invasive pole. (**E**) Initiation of chromosome replication at the invasive pole. The TLFM analysis showed that all of *B. bacteriovorus* cells after entering *E. coli* did not flip inside the prey's periplasm (n=90).

Red - PilZ-mCherry labeled cytoplasm of *B.* bacteriovorus attack-phase cell and green - DnaN-mNeonGreen of *B. bacteriovorus.* Photos represent merged bright-field and fluorescence (red

and green) images. The *B. bacteriovorus* and *E. coli* cells are marked by yellow and white dotted lines, respectively. Pictures were taken every 1 min. Scale bar = 1 µm.

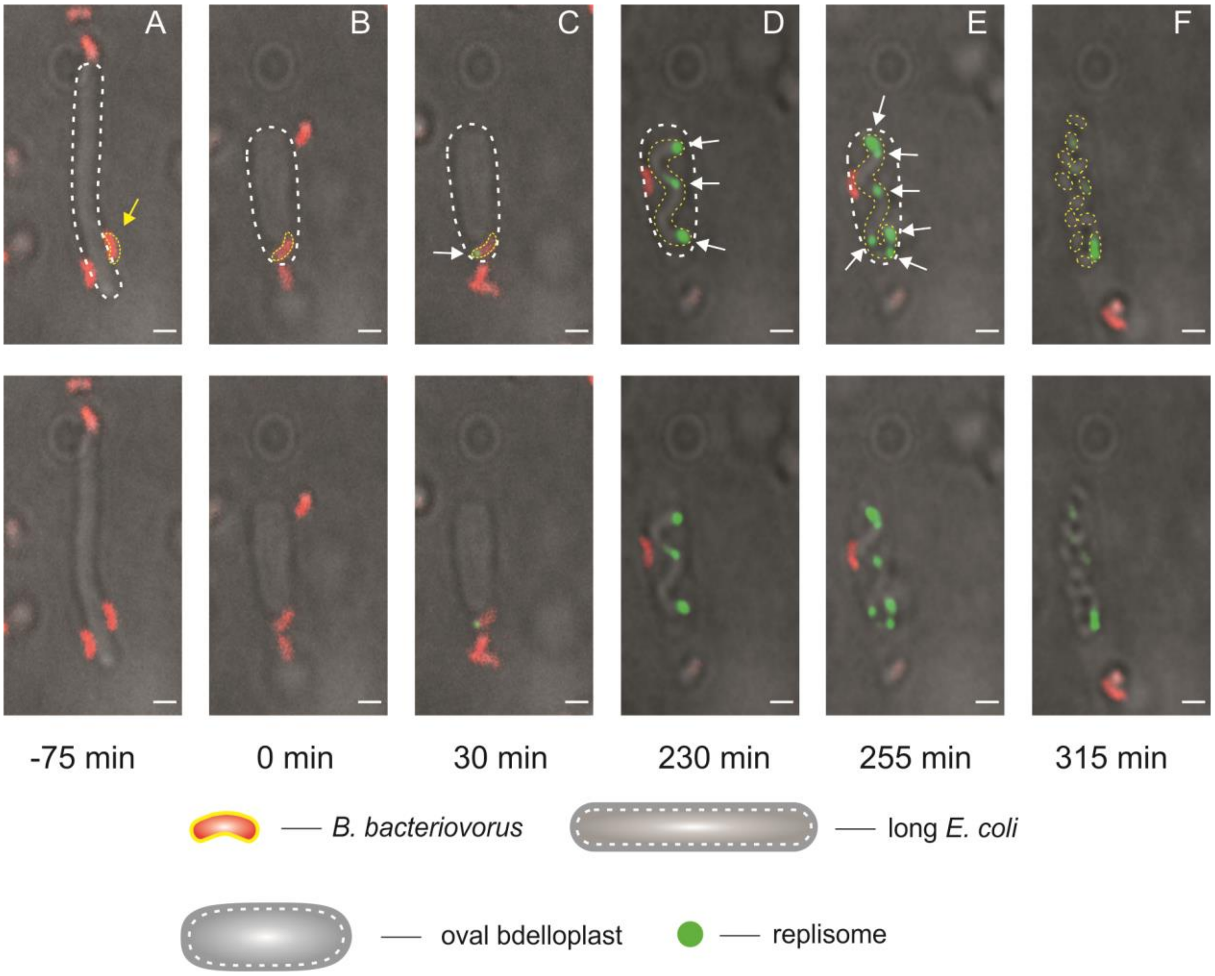

Figure S4 *B. bacteriovorus* growth and chromosome replication in an abnormally elongated *E. coli* cell

(**A**) Attachment of *B. bacteriovorus* to an elongated E. coli cell. (**B**) Bdelloplast formation contracting and rounding the large *E.coli* cell, time = 0 min. (**C**) Initiation of chromosome replication. (**D-E**) Further growth and chromosome replication. (**F**) Progeny cells. The yellow arrow highlights the single predatory cell that invades the *E. coli* cell. The white arrows indicate the positioning of replisomes in the *B. bacteriovorus* filament.

Red - PilZ-mCherry labeled cytoplasm of attack-phase *B. bacteriovorus* and green - DnaN-mNeonGreen of *B. bacteriovorus.* Photos represent merged bright-field and fluorescence (red and green) images. The *B. bacteriovorus* and *E. coli* cells are marked by yellow and white dotted lines, respectively as determined by careful analysis of bright-field images. Scale bar = 1 µm.

Supplemental Tables

Table S1 Comparison of the average time of replisomes appearing in free-living and newly-released *B. bacteriovorus* cells

	**Replisome appearance** (mean±SD) [min]				**Time intervals** (mean±SD) [min]			**Total replication time** (mean±SD) [min]
	**I**	**II**	**III**	**IV**	**I → II**	**II → III**	**III → IV**	
**Free-living cells**	74±26	133±32	164±33	177±29	59±20	32±18	27±15	144±26
**Newly-released cells**	*** 23±11	77±21	103±15	128±17	53±16	26±15	26±13	140±20

*** p-value < 0.001

Legends to Supplemental Movies

Movie S1 Time-laps imaging of *B. bacteriovorus* entry into *E. coli* periplasm in relation to invasion pole and subcellular localization of DnaN-mNeonGreen (green) in strain HD100 DnaN-mNeonGreen/PilZ-mCherry. Predatory cell indicated by PilZ-mCherry (red). Bright-field (grey) signals were taken every 1 min.

Movie S2 Time-lapse imaging of replisomes in *B. bacteriovorus*. Subcellular localization of DnaN-mNeonGreen (green) in strain HD100 DnaN-mNeonGreen/PilZ-mCherry. Predatory cell indicated by PilZ-mCherry (red). Bright-field (grey) signals were taken every 5 min.

Movie S3 Time-laps imaging of replisomes in *B. bacteriovorus* growing in abnormally elongating *E. coli* cell. Subcellular localization of DnaN-mNeonGreen (green) in strain HD100

DnaN-mNeonGreen/PilZ-mCherry. Predatory cell indicated by PilZ-mCherry (red). Bright-field (grey) signals were taken every 5 min.

Movie S4 Time-laps imaging of *B. bacteriovorus* chromosome growth and replication in two independent host cells. Subcellular localization of DnaN-mNeonGreen (green) in strain HD100 DnaN-mNeonGreen/PilZ-mCherry. Predatory cell indicated by PilZ-mCherry (red). Bright-field (grey) signals were taken every 5 min.
